# A monocyte and interferon based cell therapy for the treatment of ovarian cancer

**DOI:** 10.1186/2051-1426-3-S2-P222

**Published:** 2015-11-04

**Authors:** Daniel Green, Chase Johnson, Kathryn Zoon

**Affiliations:** 1 Cytokine Biology Section, NIAID, NIH, Bethesda, MD, USA; 2 Cytokine Biology Section, NIAID, NIH, Iowa City, IA, USA

## 

Ovarian cancer is the number one cause of death due to gynecological malignancies, and the fifth leading cause of death in cancer in women. While surgical debulking and chemotherapy cause an initial remission in disease, approximately 75% of patients will relapse. The relapse is characterized by chemotherapy refractory disease. Currently there are no definitive second line treatments for patients who fail standard of care. Patients with ovarian cancer have a 5-year survival rate of 25-30%, making it one of the most aggressive malignancies.

We have designed a new cell therapy by using the patient's innate immune system (monocytes primed with Interferon Alpha and Gamma) to kill metastatic lesions of the peritoneal cavity.

Monocytes isolated from patients with therapy resistant ovarian cancer and stimulated with interferons alpha and gamma are potent killers of tumor cell lines *in vitro*. We have also found that patient monocytes are more cytotoxic than age match healthy controls. We have shown that the major mechanism of monocyte-mediated killing is the up regulation of TRAIL on monocytes. Ovarian cancer cell death is mediated in a Caspase-8 dependent manner. Furthermore, we have shown that the non-classical (CD14^lo^CD16^+^) subset of monocytes is more cytotoxic than the classical (CD14^+^CD16^-^) subset. Finally, we have shown that this therapy is effective in a mouse model of ovarian cancer. Based upon these results we are preparing an investigational new drug (IND) with the FDA to test this therapy in human subjects.

